# Fracture of the Pelvis and Rupture of the Bladder

**Published:** 1827-05

**Authors:** 

**Affiliations:** the Middlesex Hospital.


### RUPTURED BLADDER.

### CASE I. *Fracture of the Pelvis and Rupture of the Bladder.* Treated by Mr. BELL, at the MIDDLESEX HOSPITAL.

SEPTEMBER 22d, 1826.—James Simpson, aged twenty-three; whilst employed in undermining the foundation of a wall, it fell suddenly, and buried him in the ruins. He was dug out insensible, and in this state bled, and shortly afterwards was brought to the hospital. He is pale and cold, and his pulse is small; but he is now perfectly sensible. He complains of great pain in his back, across the loins, and down the thighs; but there is no paralysis of the lower extremities, to lead to the supposition that the spinal marrow is in any way injured. There are bruises on various parts of his body, and the right testicle is lying out of the scrotum.

Twelve M.—He has not made water since the morning; and, although now feeling the desire to do so, he is unable to pass a drop. The catheter was used, and when, with great difficulty, it was fairly introduced into the bladder, nothing but clots of coagulum escaped; the urethra, at the bulb and membranous part, felt rough, and here the point of the catheter seemed to be entangled.

Cupping glasses to the loins ad ℥ xvi. Fomentations to the belly and perineum.—Pulv. Jalapæ gr. xv.; Hydr. Subm. gr. v. statim.

Ten P.M.—No urine had passed, but apparently his sufferings were not more than those of one after any common injury. On introducing the catheter, coagula, with a small quantity of bloody fluid, were evacuated. Conceiving that the instrument was blocked up by coagulated blood, the bladder was injected; and but a small quantity of the warm water returned, which however was tinged with blood. Pulse small, and the countenance haggard.

23d.—Bowels have been freely evacuated, and a small quantity of bloody urine drawn off by the catheter. As the pain in the loins continued, eighteen leeches were applied. The pulse is still weak, and the skin cold and chilly. Since his first admission, he has expressed no urgent desire to make water, and drawing it off by the catheter does not give him much relief.

At noon, the pulse rising, and the pain in the abdomen becoming more acute, he was bled to faintness, (twenty-one ounces,) and an enema of house medicine and gruel administered. He vomited some matter of a stercoraceous appearance; the sickness continued, and at night he gradually sunk, apparently without any violent pain.

*Dissection, fifteen hours after death.*—The pelvis was found to have been fractured, the fracture running from the right pecten of the pubis through the thyroid foramen. Bloody fluid in the pelvis, into the cavity of which the catheter passed, the bladder being torn from the membranous part exactly where it joins the prostate. There was some fluid in the bladder, but this viscus was not distended. None of the vertebræ were injured, but a large ulcer was found in the ileum, near the caput coli, although in appearance, the individual had been perfectly healthy. There was a little increased vascularity of the intestines, showing the commencement of inflammation.

